# Reply to the Letter from Dr. Newman

**Published:** 1879-04

**Authors:** E. C. Dudley

**Affiliations:** Chicago


### ARTICLE XVII.

### REPLY TO THE LETTER FROM DR. NEWMAN.

*Chicago*, Feb. 25, 1879.

The communication of Dr. Newman, which was read at the last meeting of the Chicago Gynæcological Society, is so remarkably colored and so misleading in what it contains and in what it strangely omits, that justice alike to Dr. Newman, to Dr. Emmet, and to the medical profession, demands a reply. Dr. Newman bases his claim upon priority of publication, and is aggrieved because the profession accords to Dr. Emmet the " credit of being the first to appreciate and describe the lesion as a cause of disease." In the paper to which Dr. Newman has alluded, published February, 1869, Dr. Emmet, in connection with the subject of dysmenorrhœa and sterility, speaks of bilateral division of the cervix as an operation productive of evil, and says :

"After this has been done (bilateral division) other difficulties of a serious character arise as a consequence, which more than counterbalance any temporary advantage. But I shall again refer to this subject when treating of lacerations following parturition."

On another page he sums up the evils arising from bilateral division of the cervix, as follows :

"My objections to the lateral operation * * * as an exciting cause of disease are due to the fact that, if gaping of the flaps occur a tendency exists to a rolling out of the lining membrane of the canal. An erosion extending into the canal is soon established, which will recur as often as healed, with the result in time that the whole organ becomes hypertrophied. This source of irritation is due to the fact that whenever the female is on her feet the flaps become separated from pressure on the posterior wall of the vagina. The everted lips in due time become softened and flattened so as to lose all appearance of ever having been in contact. I have seen this condition presenting the appearance of an enormous cervix, over two inches in diameter, with the whole organ enlarged, and every attempt at treatment proving a failure until the true condition was appreciated. I have frequently observed the same effects following lateral laceration of the neck as a result of parturition, and to some extent where only one side had been involved. * * * When lacerated laterally, and the case has been of long standing, the edges of the neck will be felt projecting beyond the vaginal junction as the top of a half-grown mushroom over its stalk. I invariably bring about union of the two surfaces before attempting any other treatment (for dysmenorrhœa, or sterility), whenever I meet this condition in practice, whether it be the result of surgical interference or a consequence of labor."

Then follows a description of the operation substantially as he performs it to-day.

It is therefore certain that Dr. Emmet described laceration as a cause of disease and its operative cure in a paper published more than two years before Dr. Newman published his memoir.

But what does Dr. Newman mean in the following comments upon the very paper from which I have just quoted.

"He (Emmet) had not, it would seem, at the time of writing that paper (February, 1869) regarded lateral laceration as a cause of disease, but simply as a lesion that interfered with method of treating dysmenorrhœa and sterility by anterior or posterior division. But on the 28th of September,

1874, * * * he, for the first time, described this lesion as a cause of disease."

The following is quoted from the memoir of Dr. Mundé,* editor of the *Americal Journal of Obstetrics.*

"Emmet's † first operation was performed November 27, 1862, in the presence of his assistant, Dr. G. S. Winston and of Dr. T. G. Thomas. Although during the next seven years he repeated the procedure many times in the presence of numerous professional gentlemen from all parts of the Union, his first published account of the operation and its technique did not appear until February, 1869. * * * In this publication Dr. Emmet was preceded by a few months by Dr. M. A. Pallen ‡ who, independently, he informs me, described substantially the same operation for the same purpose. * * Dr. Pallen calls the lesion "uterine harelip." The operation itself, and after-treatment, are described precisely as by Dr. Emmet, to whom Dr. Pallen (in a letter to me) unqualifiedly accords the priority in devising the operation and recognizing its indications. * * * In spite of these two papers and the constantly increasing number of operations performed by Dr. Emmet, amounting up to 1874 to nearly 200, it was not until the reading of a second and more lengthy paper by Dr. Emmet, * * * September, 28, 1874, * * * that the profession became fully aroused to the immense value of the discovery of the lesion, its consequences and its cure."

Dr. Pallen, who actually anticipated Dr. Emmet in publication, and who operated long before Dr. Newman pretends to have operated, lays no claim to priority in devising the operation or recognizing its indications, while Dr. Newman deliberately implies priority in publication, which he ought to know does not belong to him; and what is even more exasperating, he has left in doubt the question whether he did not also precede Dr. Emmet in recognizing laceration as a cause of disease and devising the operation for its cure, for while he shows a remarkable familiarity with the memoirs of Dr. Emmet, from which he quotes detached sentences and suppresses others to suit his purpose, he strangely omits all mention of the date of Dr. Emmet's first operation, November 27, 1862, six years before the memoir of Dr. Pallen, seven before that of Dr. Emmet, and nine before his own, but is careful to state that at the time Dr. Emmet "*First called the attention of the profession to laceration of the*

* *American Journal of Obsterics*, Jan., 1879.

† A paper by Dr. E. C. Dudley, in the CHICAGO MEDICAL JOURNAL AND EXAMINER, March, 1879, contains the full report of Emmet's first case.

‡ St. Louis *Medical and Surgical Journal*, May 10, 1868.

*cervix as an unrecognized cause of disease*," meaning 1874, not 1869, he (Newman) was treating such cases sometimes by amputation when the cervix was much diseased, and by suture when the parts were in condition, or could be brought in condition to unite.

Has Dr. Newman studied the methods of Emmet with such diligence in vain ? If so, why does he talk of amputation in the very class of cases in which, after proper preparatory treatment, the suture accomplishes its most brilliant and satisfactory results ? Experience has abundantly proved that all of these worst cases may be brought in condition for union unless some constitutional cause prevents.

But if Dr. Newman had presented his memoir at any time after 1862, he would have difficulty in establishing his claim to novelty, because since that date few weeks have passed in which Dr. Emmet failed to deliver an informal clinical lecture at the Woman's Hospital, and in these lectures laceration of the cervix was always a favorite theme. Long before the memoirs either of Newman or Pallen appeared, representatives of the profession from *remote States* in the Union, and many also from Europe, had heard him describe laceration as a cause of disease, the treatment preparatory to operation, and had seen him perform the operation. Any one by visiting the Woman's Hospital on almost any of Emmet's operation days, could therefore have carried away the material for an excellent memoir on laceration of the cervix. In fact, the memoirs of Emmet merely contain the substance of three clinical lectures in pamphlet form.

Dr. Emmet never claimed to have been the first to recognize laceration of the cervix. I have his written statement, in a recent letter, that the "cleft" or "fissure" or "uterine harelip" must have been felt and seen from the beginning, but he was the very first to demonstrate that the erosion of the everted intracervical membrane, formerly called "ulceration," is always a laceration. No one before him showed that the cervix may present a normal shape and an apparently normal os externum and yet be the seat of laceration extending even past the utero vaginal attachment. He first demonstrated that the lacerated flaps generally become so softened and flattened by resting on the floor of the pelvis that they lose all appearance of ever having been in contact with one another, and that the intra-uterine tissue and membrane roll out and form a spurious external cervix which, in shape and position, usurps the place of the normal external cervix. As the late Dr. Peaslee* said, "it was he, who in a happy moment brought the anterior and posterior surfaces together with two tenacula, and instantly (by causing the everted tissue and membrane to roll in) demonstrated that what we all thought was an ulceration was nothing more or less than a laceration.

Dr. Emmet is falsely represented as urging as a condition of success, "that the opposite edges of the laceration be quite thoroughly pared or cut away. He never advocated such denudation except in a few very exceptional cases. Dr. Newman will probably, therefore, have in future little or no use for his idea of the "conical cervix" as a result of the operation. The correspondent urges some *a priori* objections to the operation, but with commendable modesty assigns his limited experience as a reason for not insisting upon them all.

When Dr. Newman proves that laceration occurs more frequently in premature than in full term delivery he will have no difficulty in establishing the accuracy of his assertion. But respecting these opinions I have nothing to add, since this is a controversy, not of opinions, but of facts.

No amount of prospective fame ever tempted Dr. Emmet into premature publication. He waited until he had operated two hundred times for laceration of the cervix, and then twelve years after his first operation so exhausted the subject that little remains to be said. In his work on Diseases of Women, now in preparation and soon to be published, he has pursued a similar course, for few, if any, surgeons in the past twenty-five years have done more of modern gynæcological surgery than he has done. The chaff, the controversies, and out-worn theories which beset the practitioner and distress the student are chargable to men who have written for reputation or notoriety rather than from experience, reflection, and classified knowledge.

E. C. DUDLEY, M.D.

* Mundé *Am. Jour. Obst.*, Jan., 1879.

